# Inter-hospital transfer: the crux of the trauma system, a curse for trauma registries

**DOI:** 10.1186/1757-7241-18-15

**Published:** 2010-03-16

**Authors:** Hans Morten Lossius, Thomas Kristiansen, Kjetil G Ringdal, Marius Rehn

**Affiliations:** 1 Department of Research, Norwegian Air Ambulance Foundation, Drøbak, Norway; 2 Department of Surgical Sciences, University of Bergen, Bergen, Norway; 3 Faculty of Medicine, Faculty Division Oslo University Hospital, Ullevål, University of Oslo, Oslo, Norway

## Abstract

The inter-hospital transfer of patients is crucial to a well functioning trauma system, and the transfer process may serve as a quality indicator for regional trauma care. However, the assessment of the transfer process requires high-quality data from various sources. Prospective studies and studies based on single-centre trauma registries may fail to capture an appropriate width and depth of data. Thus the creation of inclusive regional and national trauma registries that receive information from all of the services within a trauma system is a prerequisite for high quality inter-hospital transfer studies in the future.

## Commentary

In a recent article published in the Scandinavian Journal of Trauma, Resuscitation and Emergency Medicine, Professor Katsaragakis and colleagues depict patient flow through what they describe as a non-trauma-system setting in Greece [1]. Their study contributes to a growing body of inter-hospital transfer studies and provides an opportunity to comment on the complexity of analyzing trauma transfer.

The development of a dedicated trauma system to deal effectively with severely injured patients was initiated in the early 1980's, with the American College of Surgeons (ACS) playing a leading role [2]. The trauma system, as described by the ACS, is a purposeful organisation of health care resources that ensures the optimal treatment of injured patients [3]. *Inclusive *trauma systems define roles for all levels and types of health care facilities and personnel that provide care for trauma patients from the scene of injury to rehabilitation. During the last decades of the 20th century, several studies reported increased survival rates after the creation of such dedicated trauma systems [4]. A number of European countries are adapting these principles, and networks of trauma hospitals are evolving [5-7].

The demand for cost reduction and centralisation of advanced health care services has lead to a shift of specialist resources and severely injured patients away from local hospitals towards regional centres and university hospitals. The local hospital has become a potentially hazardous diversion for major trauma patients, thereby necessitating safe and efficient pre-hospital triage and inter-hospital transfer procedures.

Organised trauma systems with dedicated trauma centres ensure (at least in theory) that patients in need of specialist resources are brought directly to an appropriate level of care. However, not all injured patients should be brought directly to a trauma centre, and the quality of care prior to reaching the trauma centre may have significant impact on patient outcome [8]. Despite trauma system implementation, secondary transferrals remain a significant proportion of the trauma population [3]. Several intentional as well as non-intentional reasons for inter-hospital transfer exist: suboptimal pre-hospital diagnostic capacity causing unnecessary transport to local hospitals, patients in need of urgent stabilization before final transport is feasible, or local hospital functioning as a rendezvous point for retrieval services.

Throughout the logistically complicated inter-hospital transfer, the patients' wells being relies on optimal inter-disciplinary communication, cooperation and transition of care. The intended positive effect of dedicated trauma systems on patient outcomes might vanish due to suboptimal triage or a lack of routines and competence causing unfavourable treatment delays. Consequently, the inter-hospital transfer process is crucial to the system's efficiency and should be investigated accordingly. The development of performance measures for emergency medical systems have been called for and the appropriateness of triage and transfer processes has been suggested as quality indicators of trauma systems [9,10].

The North-American trauma research has evolved from expert panel studies, towards trauma registry-based analyses and population-based studies. Administrative registries are designed for purposes other than research and have been criticised due to their lack of important clinical information [4]. Trauma researchers, therefore, often rely on evidence generated through registry-based observational studies. Specialised trauma centre registries have been developed and attempts are made to standardize variables and definitions included in these registries [11]. However, quality assurance of the regional trauma systems require data collection beyond the sum of information gathered at individual trauma centres [12].

To assess the management of patients who are transferred within a trauma system and to compare their outcomes with other groups of patients without selection bias, investigators must have extensive access to information. Data must be collected from the pre-hospital emergency medical services, the local hospitals, the transferring units and the receiving trauma centres within a region. Further, investigators must be able to track individual patients through these various components that make up the trauma system. A single-centre based trauma registry will therefore struggle to provide all necessary data, making investigators dependent on additional data collection. The aforementioned study from Greece illustrates this limitation. The study is based on prospectively gathered data that was collected to assess the feasibility of developing a national Greek trauma registry [13]. With 40% of the trauma receiving hospitals in Greece participating in the registry, a large number of patients were transferred either from or to non-participating hospitals. Excluding these patients will reduce the completeness when attempting to map the patient-flow through the Greek trauma services. However, the information collected on patient injuries and outcomes from non-participating hospitals may be highly heterogeneous and the quality of the collected data may be questioned.

To our knowledge there are few examples of studies that successfully avoided these limitations. In Oregon, a state-wide trauma registry allowed a population-based study of survival as a function of being transferred to higher level of care [14]. In the Australian state of Victoria, a system- and state-wide registry has allowed detailed population-based epidemiological and quality improvement studies [15]. However, the investigation of inter-hospital transfers in this trauma system required additional data collection [16]. Studies on inter-hospital transfer require that data be collected from a majority of the services that make up a trauma system. The feasibility of doing this prospectively may therefore limit the extent of the studies conducted. In addition, an unstructured *ad hoc *documentation process may lead to an unacceptable quality of the gathered data.

So, we are back to the established trauma registries. Using the terms of Dreyer and Garner [17], we would argue that trauma management and inter-hospital transfers are "real-world" events whose further study requires the robust evidence provided by trauma registries. Though few existing registries have the appropriate infrastructure to allow patients to be tracked throughout the entire trauma system, the creation of such regional or national inclusive trauma registries is an absolute necessity. To improve data collection, regional and national registries must have uniform inclusion criteria, clinical variables and definitions, as well as a core set of defined performance/quality indicators [18]. The variables must include specific parameters that allow individual patients to be completely tracked throughout the trauma system. Complete data capture may, however, only be possible if the regional or national jurisdiction mandates participation by all hospitals [19] and accompanies this mandate by sufficient funding. Regional and national trauma registries could subsequently collect data to assess the appropriateness, timeliness, as well as costs and outcome of transporting patients between hospitals. The results from such assessment may serve as a crucial quality indicator of the maturity and efficiency of a trauma system. However, until such inclusive trauma registries are further developed, the analysis of inter-hospital transfer will remain a challenge for investigators.

## Competing interests

The authors declare that they have no competing interests.

## Authors' contributions

HML has had the original idea for the article and has been responsible for the overall outline of the manuscript. HML, TK, MR and KGR have all contributed with literature search as well as original and independent parts of the manuscript. TK has revised and submitted the manuscript. All authors have proof read and accepted final draft of the manuscript.
